# Supplementary material for: Open architecture of archaea MCM and dsDNA complexes resolved using monodispersed streptavidin affinity CryoEM
Source: Nat Commun. 2024 Nov 27;15:10304. doi: 10.1038/s41467-024-53745-w (PMC11603195; doi:10.1038/s41467-024-53745-w)
Supplement: Supplementary file 1 — Supplementary Information [file 41467_2024_53745_MOESM1_ESM.pdf]

Supplementary Table 1. Comparison of features from other published MCM structures. The distances were measured at the main chains of the structures.

Source		Complex	MCM layer	Resolution (Å)	PDB	Closed/ open	Spiral of hexamer	Spiral of H2I and PS1	Center channel (CTD)	Opening gate	Nucleotide	DNA
**Archaea**	*Pyrococcus furiosus* NTD	MCM	Single hexamer	3.2	4POG	Closed	---	---	---	---	---	ssDNA
	Methanopyrus kandleri		single subunit	1.9	3F8T	---	---	---	---	---	---	---
	Sulfolobus solfataricus		Single hexamer	1.86	6MLL	Closed	---	Right-handed 10 Å	20 Å	---	ADP-BeF3: 3, ADP: 3	ssDNA
			Single subunit	4.35	3F9V	---	---	---	---	---	---	---
	Sulfolobus solfataricus NTD		Single hexamer	2.8	2VL6	Closed	---	---	---	---	---	---
			Dimer	2.0	6WNZ	---	---	---	---	---	---	---
**Eukaryon**	Yeast	ORC-MCM	Single hexamer	4.4	6RQC	Closed	---	Complex	20 Å	---	---	dsNDA
		ORC-Cdc6-Cdt1-MCM		3.9	5V8F	Semi-open	No spiral	Left-handed 10 Å	25 Å	---	ATPγS: 4	dsDNA
				10.0	6WGI						---	
				7.7	6WGF	Open	Left-handed 25-32 Å	Left-handed 32Å	35 Å	6 Å	---	---
				8.1	6WGG						---	---
		MCM (phosphorylated)	Double hexamer	4.77	6F0L	Closed	---		25 Å	---	---	dsDNA
		MCM		3.8	3JA8						ADP: 6	---
				3.9	5BK4						ADP: 4	dsDNA
				3.2	7V3U						ATPγS: 5 ADP: 1	---
		MCM-DDK		3.3	7P5Z						ATP: 1 ADP: 4	dsDNA
				3.2	7PT6						ATPγS: 4 ADP: 2	---
				3.8	7PT7						ADP: 6	---
				2.9	7V3V						ATPγS: 5 ADP: 1	---
		Cdc45-MCM (NTD)-GINS	Single hexamer	3.7	3JC6				---		Apo	---
		Cdc45-MCM-GINS		4.7	3JC5				25 Å		Apo	---
				4.8	3JC7						Apo	---
				4.9	5U8T						---	ssDNA
				6.1	5U8S						ATP: 3	Forked DNA
				3.7	6RAW						ATP: 3 ADP: 3	
				3.99	6RAX						ATP: 3 ADP: 3	
				4.28	6RAY						ATP: 4 ADP: 2	ssDNA
				4.46	6RAZ						ATP: 4 ADP: 2	Forked DNA
				3.9	6U0M						ATP: 3	ssDNA
		Cdc45-MCM-GINS-Pol ε		4.98	6HV9						ATPγS: 2	Forked DNA
			Double hexamer	3.3	7QHS						ATP: 4 ADP: 2	dsDNA
		Cdc45-MCM-GINS-Csm3-Tof1-Ctf4	Single hexamer	3.7	6skl						AMPPNP: 3	Forked DNA
		Cdc45-MCM-GINS-Csm3-Tof1-Ctf4 (show only MCM N-terminal)		3.4	6SKO						AMPPNP: 5	
		Replisome:Pol a-primase		3.5	8B9A						AMPPNP: 4	Forked DNA
				3.5	8B9B							
				4.6	8B9C							
	Human			3.4	8B9D						AMPPNP: 3	
		Cdc45-MCM-GINS		3.29	6XTX						ATPγS: 3 ADP: 2	Forked DNA
		Cdc45-MCM-GINS-AND-1		6.77	6XTY						---	---
	Caenorhabditis elegans	DNSN-1_CMG_TIM-1_TIPN-1		3.75	8OUW						AMPPNP: 4	Forked DNA

Supplementary Table 2. CryoEM analysis for MCM particle distribution on mspSA affinity grids.

Screening of samples on SA grids	**MCM-apo**		**MCM-ATP-dsDNA**	
	0.05 mg/ml biotinylated	0.05 mg/ml	0.07 mg/ml biotin-tagged DNA	0.07 mg/ml
Microscope	Glacios	Glacios	Glacios	Glacios
Voltage (keV)	200	200	200	200
Detector	Falcon3	Falcon3	Falcon3	Falcon3
Å/pixel	2.0	2.0	2.0	2.0
Defocus range (μm)	-3 to -6	-4 to -7	-2 to -8	-3 to -6
Total Dose (electrons/Å^2)	50	50	50	50
Number of images from random squares	12	13	21	31
Number of particles	4,154	98	5,407	699

Supplementary Table 3. CryoEM data collection, refinement and validation statistics

**Samples**	MCM-apo	MCM-ATP-dsDNA
	0.08 mg/ml biotinylated	0.07 mg/ml biotin-tagged DNA
**Data collection and processing**		
Voltage (kV)	300	300
Detector	Gatan K3	Falcon 4
Energy filter	bioquantum<br>20 eV slit	Selectris X<br>20 eV slit
Electron exposure (e/Å^2)	40	39
Defocus range (μm)	-0.5 to -3.0	-0.5 to -3.0
Pixel size (Å)	1.072	0.932
Symmetry imposed	C1	C1
Number of frames	40	40
Number of images	4,003	14,584
Final particle images	445K	127K
Map resolution (Å)	3.26	3.57
FSC threshold	0.143	0.143
Local resolution (4 middle subunits)	2.5-3.0	3.0-3.5
**Refinement**		
Initial model used	AlphaFold2 predicted	AlphaFold2 predicted
Model resolution (Å)	3.37	3.57
FSC threshold	0.5	0.5
Map sharpening *B* factor (Å^2)	111.5	109.4
Model composition		
Non-hydrogen atoms	26842	26522
Protein residues	3753	3840
Ligands		ATP: 2, ADP: 3, MG: 3
B factors (Å^2)		
Protein	47.92	104.29
Ligand		43.24
R.m.s. deviations		
Bond lengths (Å)	0.006	0.002
Bond angles (°)	0.718	0.538
Validation		
MolProbity score	1.45	1.63
Clashscore	4.89	9.75
Poor rotamers (%)	0.41	0.37
Ramachandran plot		
Favored (%)	96.81	97.37
Allowed (%)	3.19	2.52
Disallowed (%)	0.00	0.11
Accession codes	EMD-38109<br>PDB 8X7T	EMD-38111<br>PDB 8X7U

Supplementary Table 4. CryoEM data collection, refinement and validation statistics

**Samples**	ScPol II EC	CA
	0.1 mg/ml biotinylated	0.07 mg/ml biotinylated
**Data collection and processing**		
Voltage (kV)	300	300
Detector	Gatan K3	Gatan K3
Energy filter	bioquantum<br>20 eV slit	bioquantum<br>20 eV slit
Electron exposure (e/Å^2)	50	50
Defocus range (μm)	-1.0 to -2.4	-1.0 to -2.4
Pixel size (Å)	0.829	0.829
Symmetry imposed	C1	D6
Number of frames	50	50
Number of images	1,500	11,129
Final particle images	146,186	117,411
Map resolution (Å)	2.98	3.14
FSC threshold	0.143	0.143
Local resolution	3.0-5.0	3.0-4.2
**Refinement**		
Initial model used	8TVY	8TY6
Model resolution (Å)	3.29	3.31
FSC threshold	0.5	0.5
Map sharpening *B* factor (Å^2)	72.7	137.9
Model composition		
Non-hydrogen atoms	27832	19716
Protein residues	3392	2628
Ligands	8	
Nucleotides	45	
B factors (Å^2)		
Protein	79.97	57.04
Ligand	63.90	
Nucleotides	106.93	
R.m.s. deviations		
Bond lengths (Å)	0.004	0.004
Bond angles (°)	0.950	0.993
Validation		
MolProbity score	1.83	1.62
Clashscore	9.74	8.89
Poor rotamers (%)	0.07	0.00
Ramachandran plot		
Favored (%)	95.45	97.24
Allowed (%)	4.52	2.76
Disallowed (%)	0.03	0.00
Accession codes	EMD-61287<br>PDB 9JA1	EMD-61286<br>PDB 9JA0

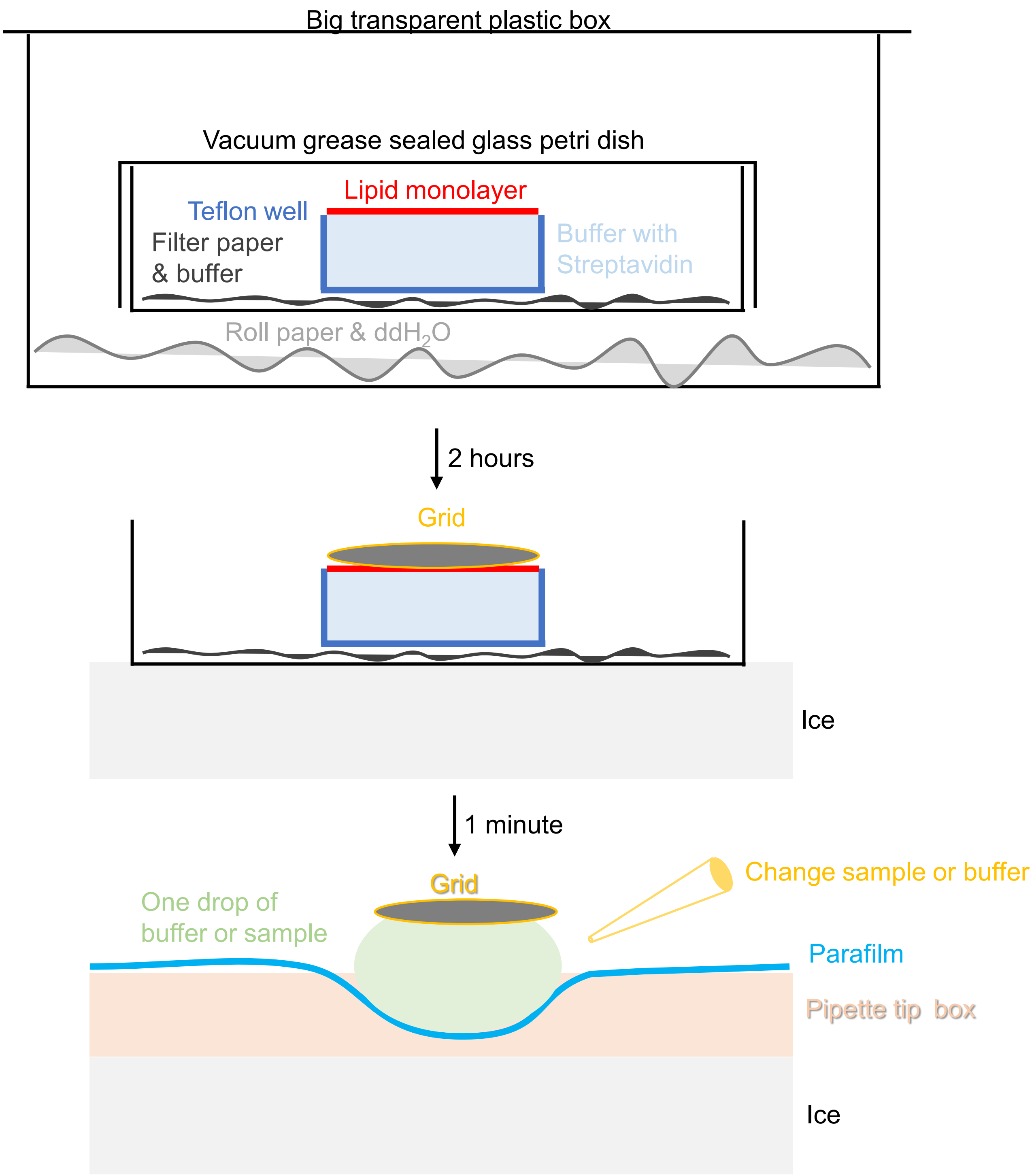

Supplementary Figure 1 | Key steps of preparing SA affinity grid. SA is incubated under the lipid monolayer for 2 hours. The grid (carbon side facing downward) is float on the sample droplet and buffer change needs to be gentle.

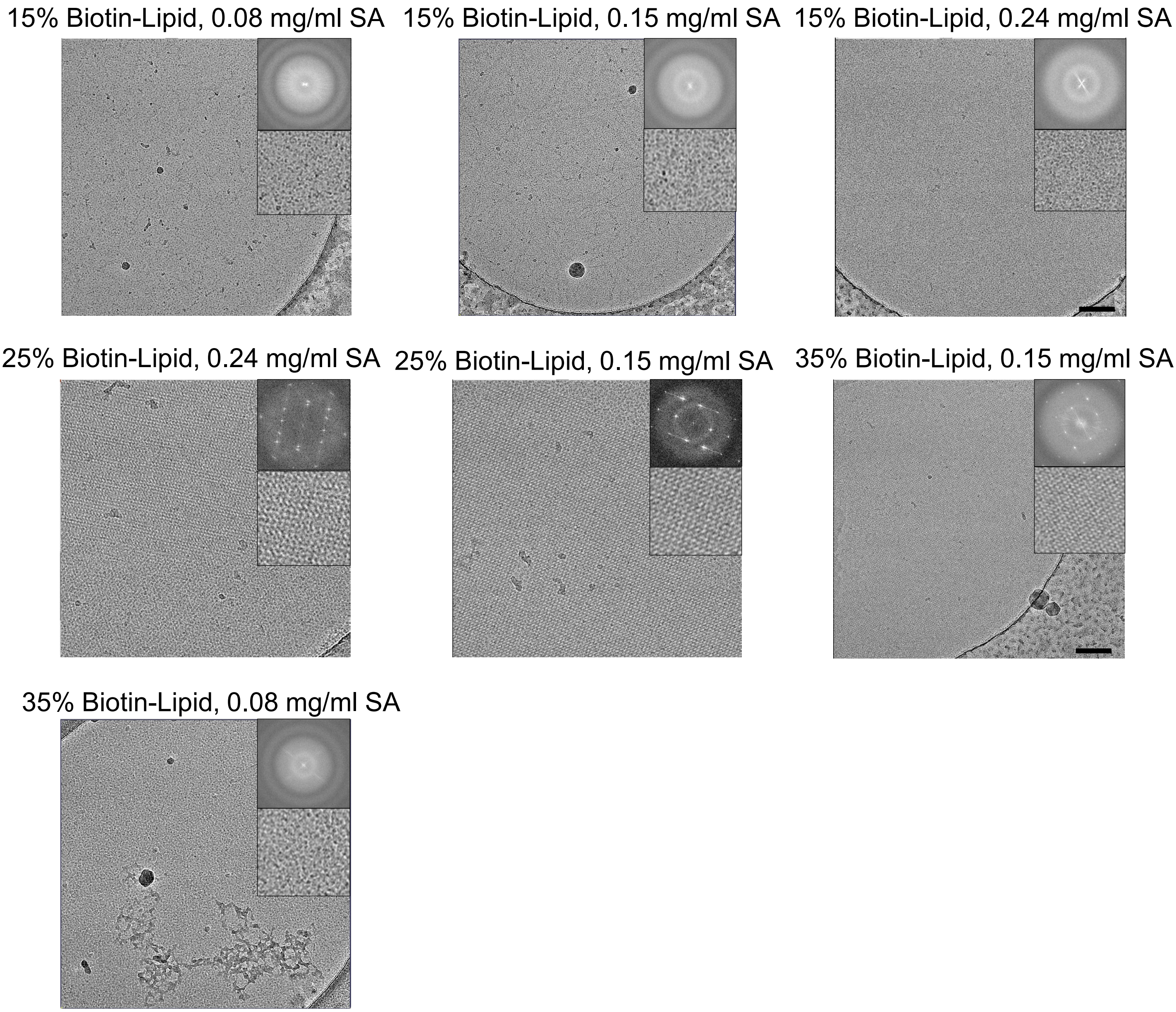

Supplementary Figure 2 | Formation of monodispersed single particle and 2D crystalline SA at the indicated conditions. Shown are representative cryoEM micrographs. Insets show the Fourier transform of the image and an enlarged view. Images are on the same scale. Scale bars, 100 nm. The experiment was repeated at least three times independently with similar results.

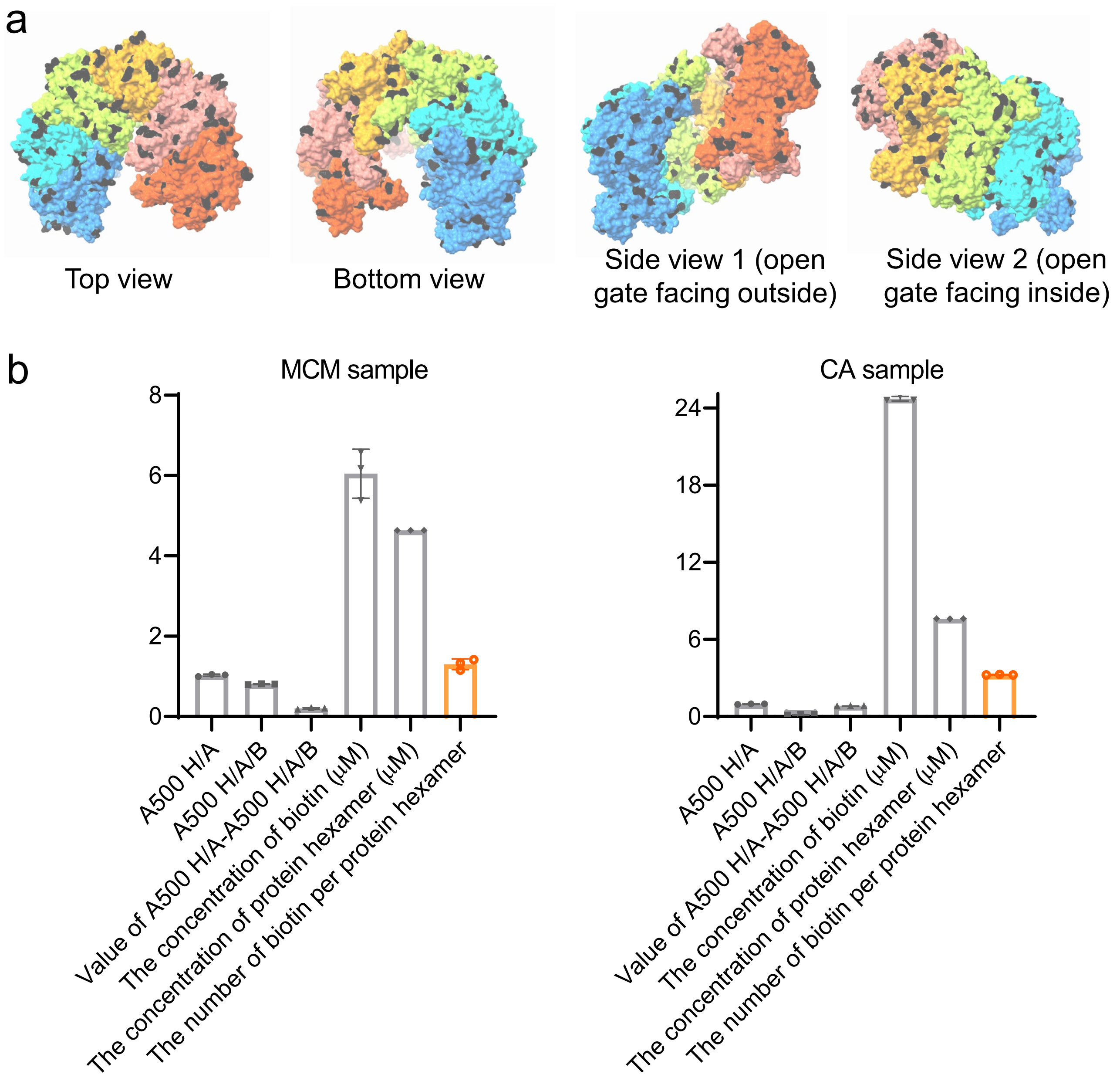

Supplementary Figure 3 | Biotinylation of protein surface lysine residues. (a) The exposed lysine residues in the structure of MCM**.** The lysine residuals are colored in black. (b) The extend of biotinylation for the MCM (left) and HIV-1 CA hexamer.

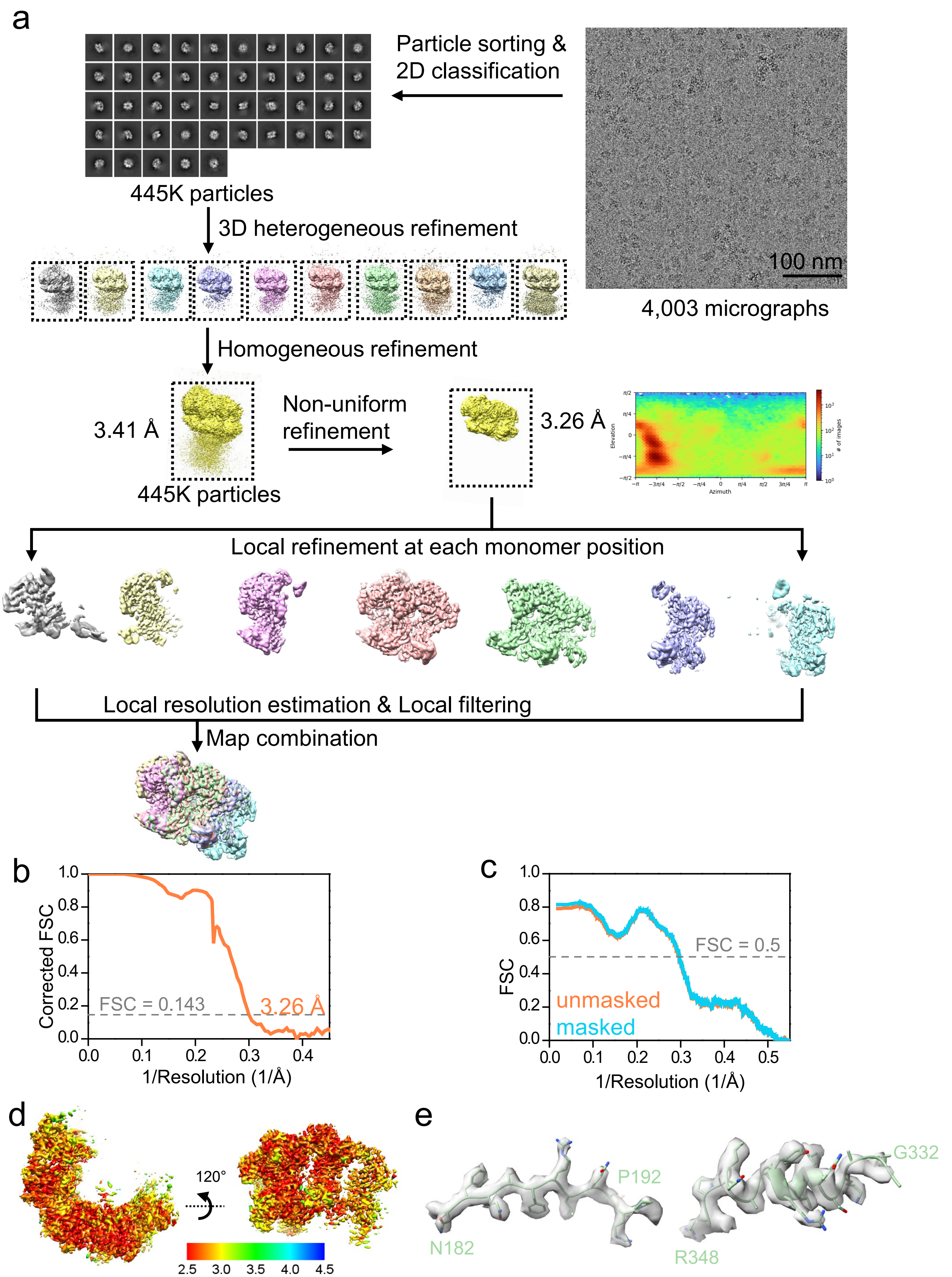

Supplementary Figure 4 | Data processing summary of MCM-apo captured by mspSA affinity grids. (a) Workflow of MCM-apo structure determination. **(b)** Corrected FSC and local resolution of the density map of MCM-apo. **(c)** Map-to-model FSC curves from Phenix. **(d-e)** Local resolution map from a central section (**d**) and representative filtered density map from CryoSPARC overlapped with the refined atomic model (**e**).

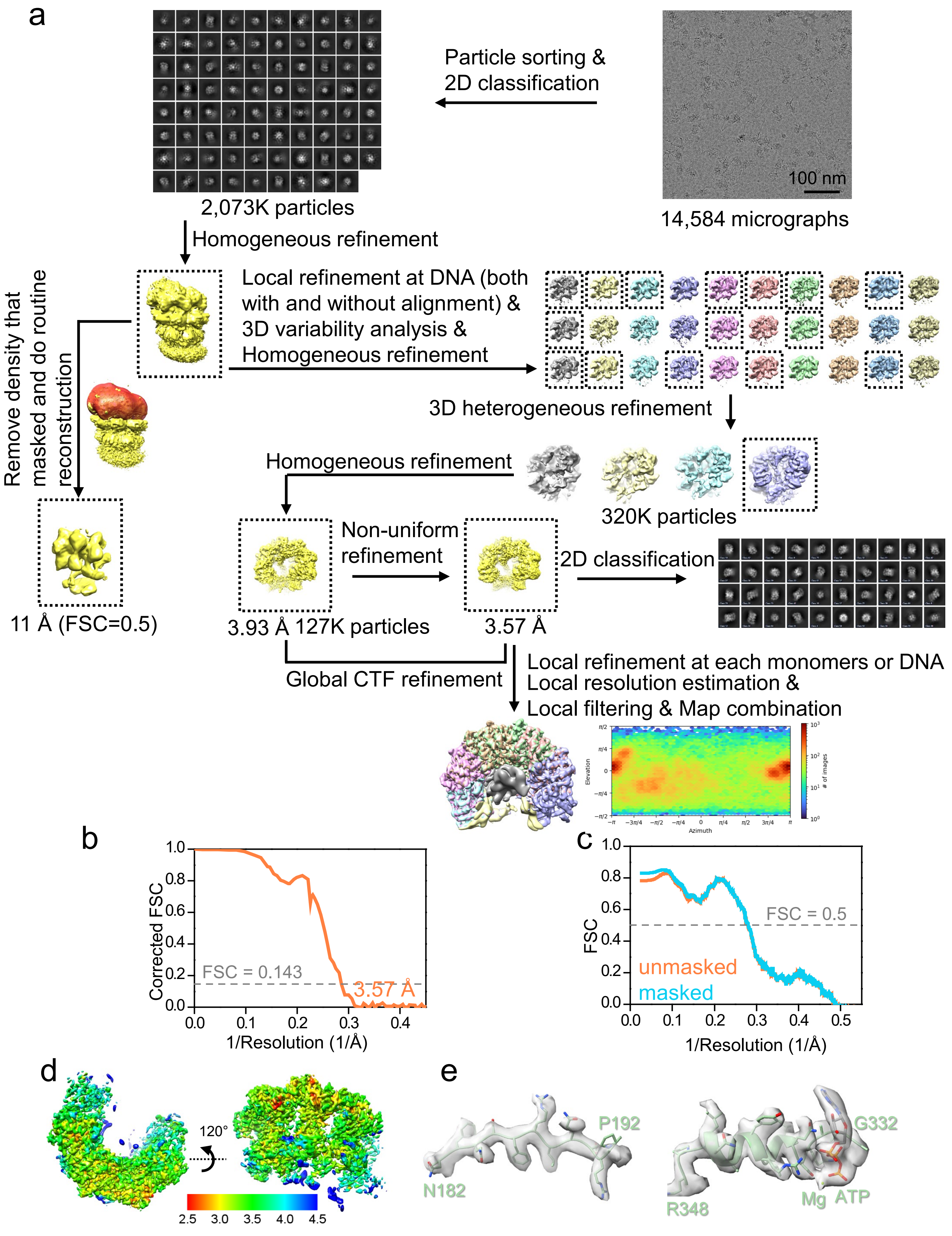

Supplementary Figure 5 | Data processing summary of MCM-ATP-dsDNA captured by mspSA affinity grids. **(a)** Workflow of MCM-ATP-dsDNA structure determination. **(b)** Corrected FSC and local resolution of the density map of MCM-ATP-dsDNA. **(c)** Map-to-model FSC curves from Phenix. **(d-e)** Local resolution map from a central section (**d**) and representative filtered density map from CryoSPARC overlapped with the refined atomic model (**e**).

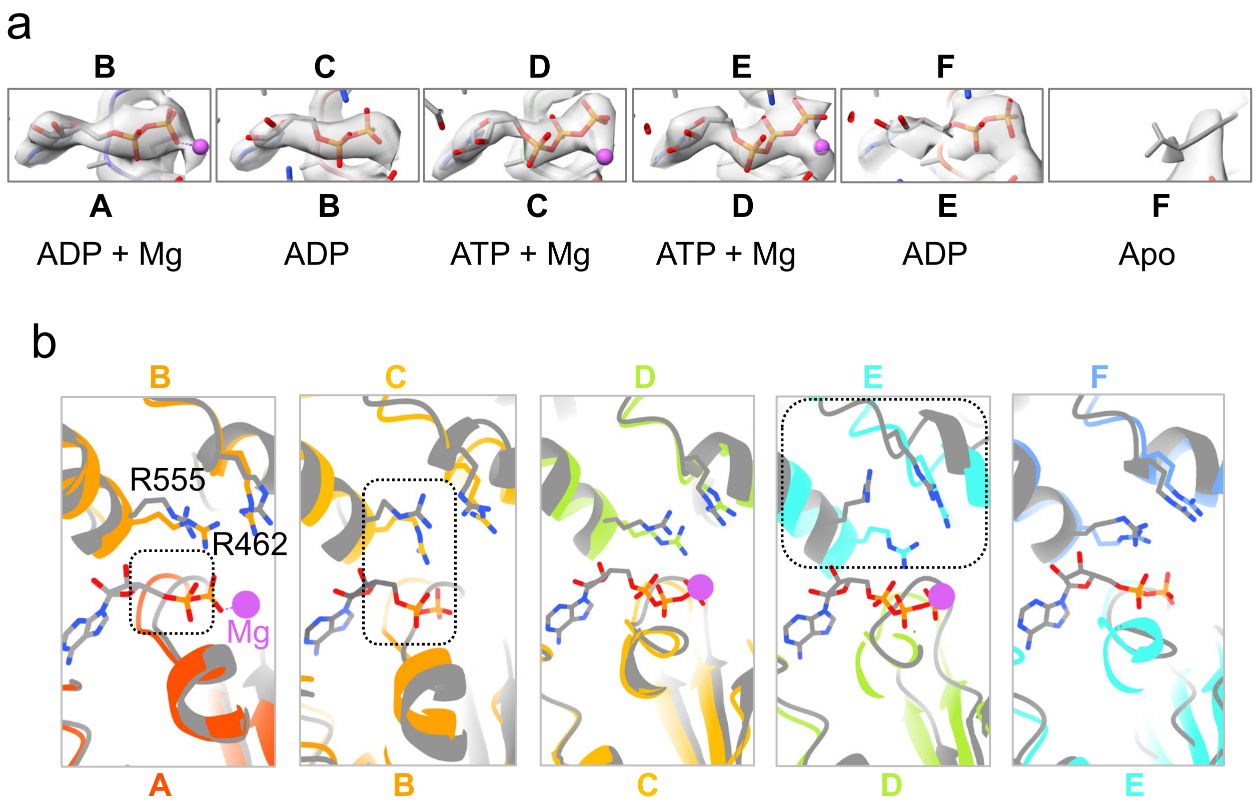

Supplementary Figure 6 | Structures of the ATP pocket in MCM-ATP-dsDNA complex. (a) Model building of ATP/ADP at the *cis* interfaces of ATP pocket. The ATP/ADP and most side chains are clearly resolved in A-E subunits, but poorly defined in F subunit. The Mg ions (purple) are visualized in A-B, C-D and D-E pockets. **(b)** Comparison of the ATP pockets between MCM-apo (grey) and MCM-ATP-dsDNA (colored). The structures were aligned on the segments 333-350 and 475-490, which are around the ATP pockets. Mg ions are shown in purple.

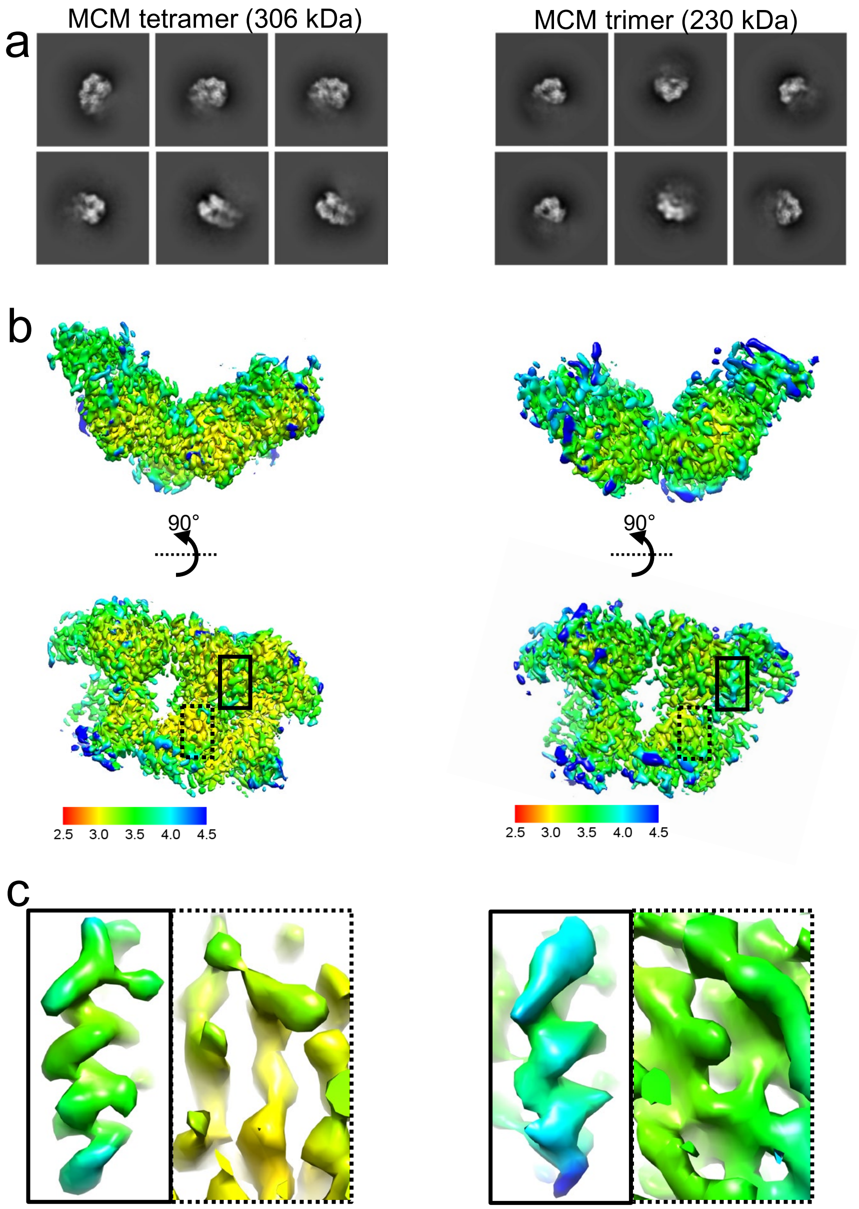

Supplementary Figure 7 | Reconstruction of subcomplexes of MCM-apo captured by mspSA affinity grids. **(a)** 2D averages of the subtracted tetramer (left) and trimer (right) MCM subcomplexes. **(b)** The overall 3D map of the subcomplexes colored according to local resolution. **(c)** Alpha helix and beta strands from each subcomplexes are shown, colored according to local resolution.

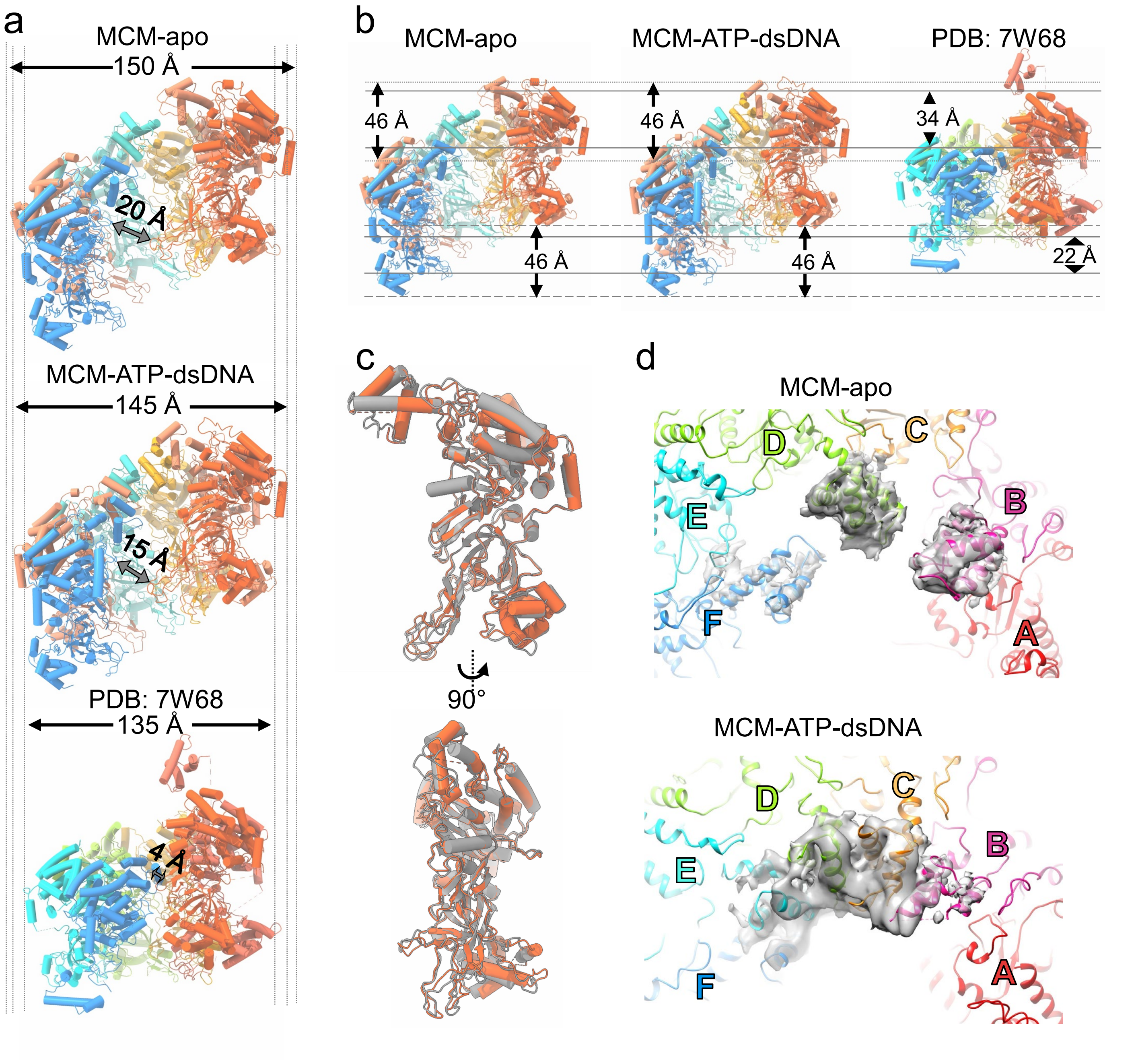

Supplementary Figure 8 | Structure comparison of MCM at open conformations. MCM-apo, MCM-ATP-dsDNA and previously reported open conformation at highest resolution 4.4 Å (PDB: 7W68) are included. **(a)** Comparison of the size of the hexamer ring and the gate among the three open MCM structures. **(b)** Comparison of the spiral rise among the three open MCM structures. **(c)** Overlay of the B subunits from MCM-apo (grey) and MCM-ATP-dsDNA (orange). The RMSD is 0.98 Å. **(d)** Comparison of the WH (grey density) between MCM-apo and MCM-ATP-dsDNA.

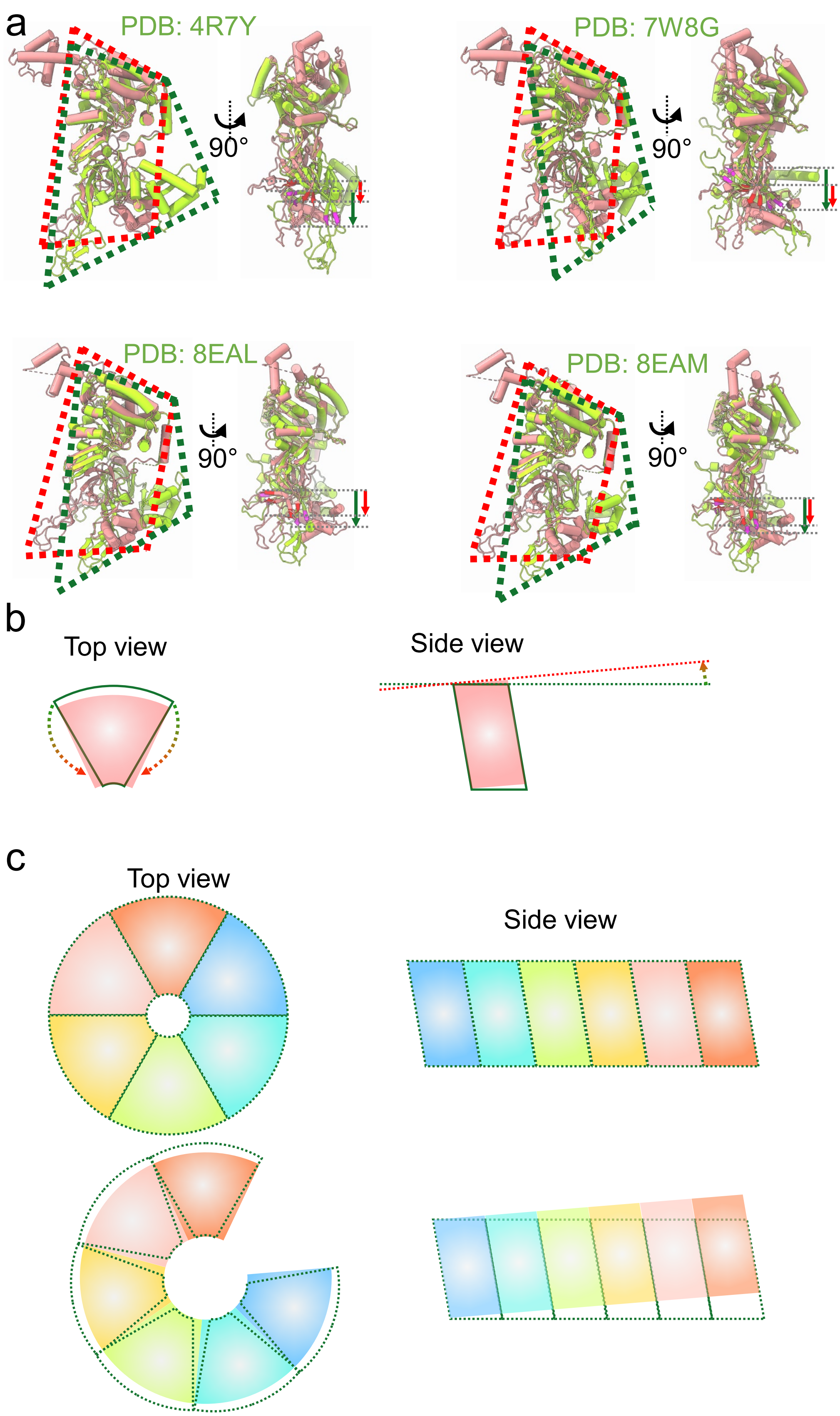

Supplementary Figure 9 | Handedness analysis of the MCM. **(a)** The B subunit of MCM-apo (pink) superimposed with the B subunit of other published MCM structures (green) aligned on CTD. The dashed lines are the outlines of the B subunits of MCM-apo structure (red) and of previous reported structures (green). Arrows indicate the height differences of NT and ZF in a single subunit. **(b)** A simplified shape representation of MCM B subunit, in top and side views. **(c)** A simplified shape model of MCM hexamer in the close conformation (top, from previous structures) and in the open conformation (bottom, this study), shown in top (left) and side (right) views. The side views are viewed from the center channel. The handedness and the size of central channel are clearly illustrated with the model.

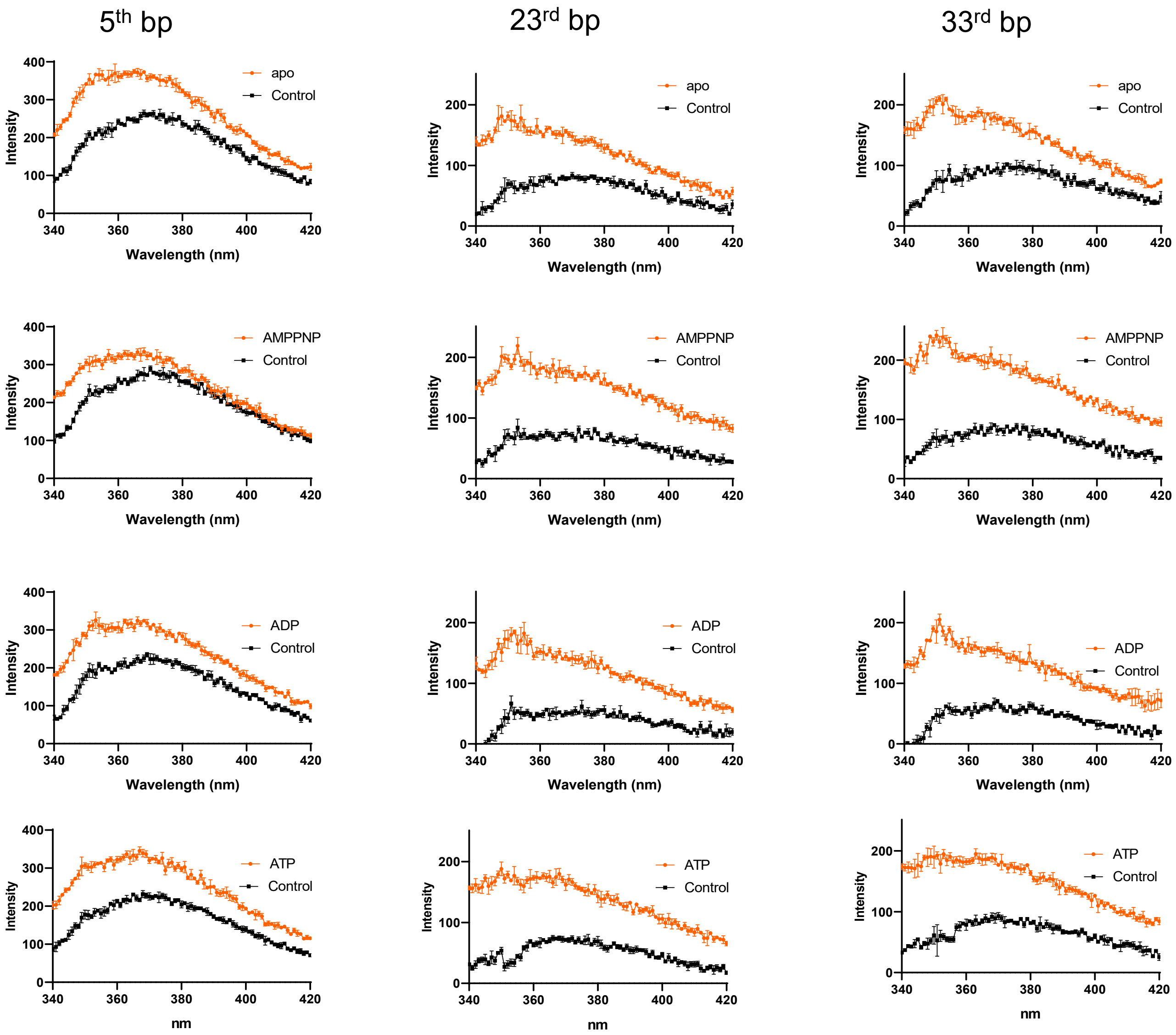

Supplementary Figure 10 | Emission spectrums of the 2-AP labeled DNA. The error bars are the standard deviation of the meareements (N=3).

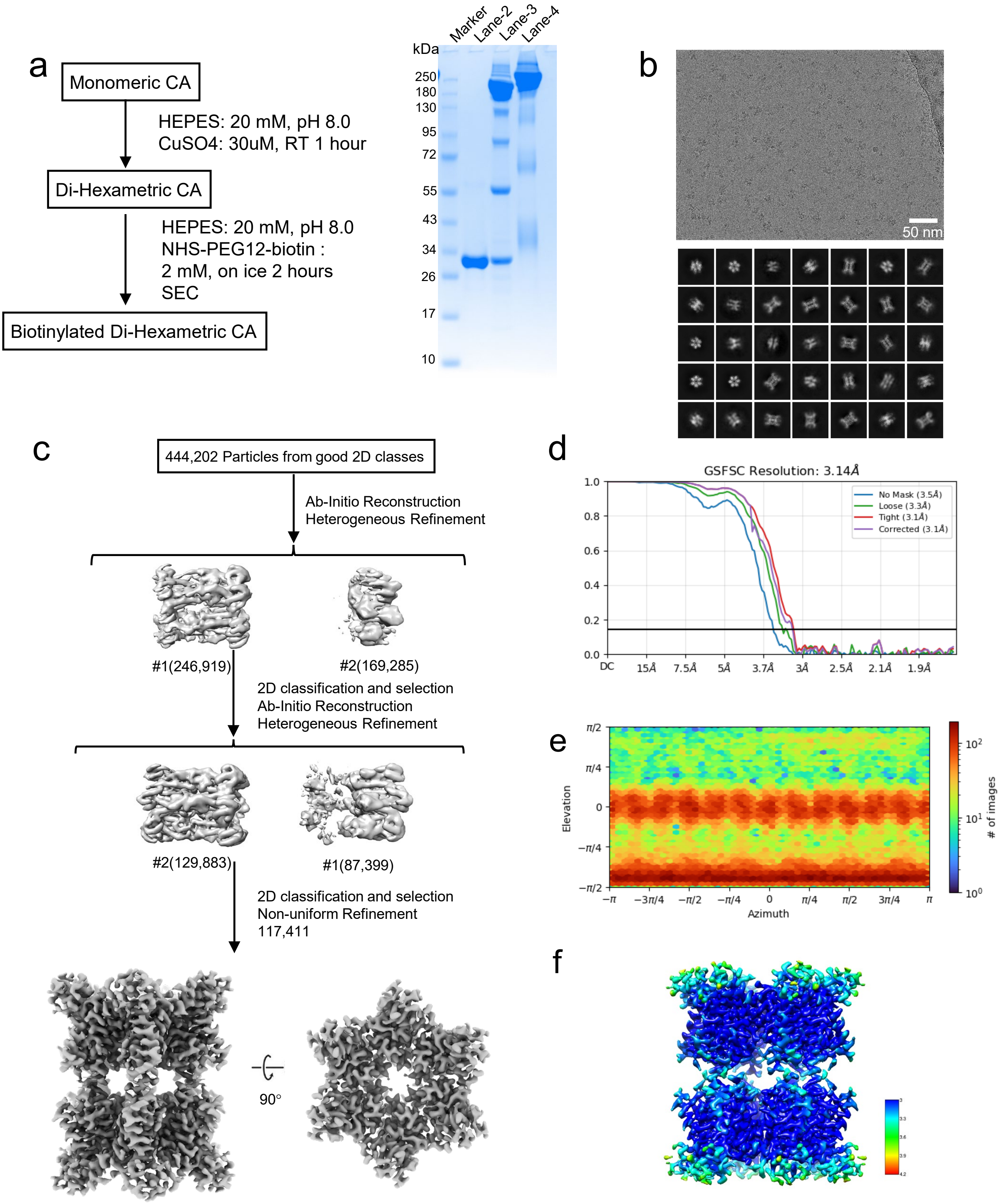

Supplementary Figure 11 | Data processing summary of HIV CA Di-hexamer captured by mspSA affinity grids. (a) HIV CA Di-hexamer protein generation and SDS-PAGE analysis of monomeric CA, CuSO4 digested CA and biotinylated Di-hexamer after SEC in lane-2, lane-3 and lane-4 respectively. **(b)** Rrepresentative raw micrograph (top) and gallery of reference-free 2D class averages (bottom) of CA Di-hexamer. **(c)** Workflow of CA Di-hexamer structure determination. **(d-f)** Corrected FSC **(d)**, orientational distribution heat map **(e)** and local resolution **(f)** of the density map of CA Di-hexamer.

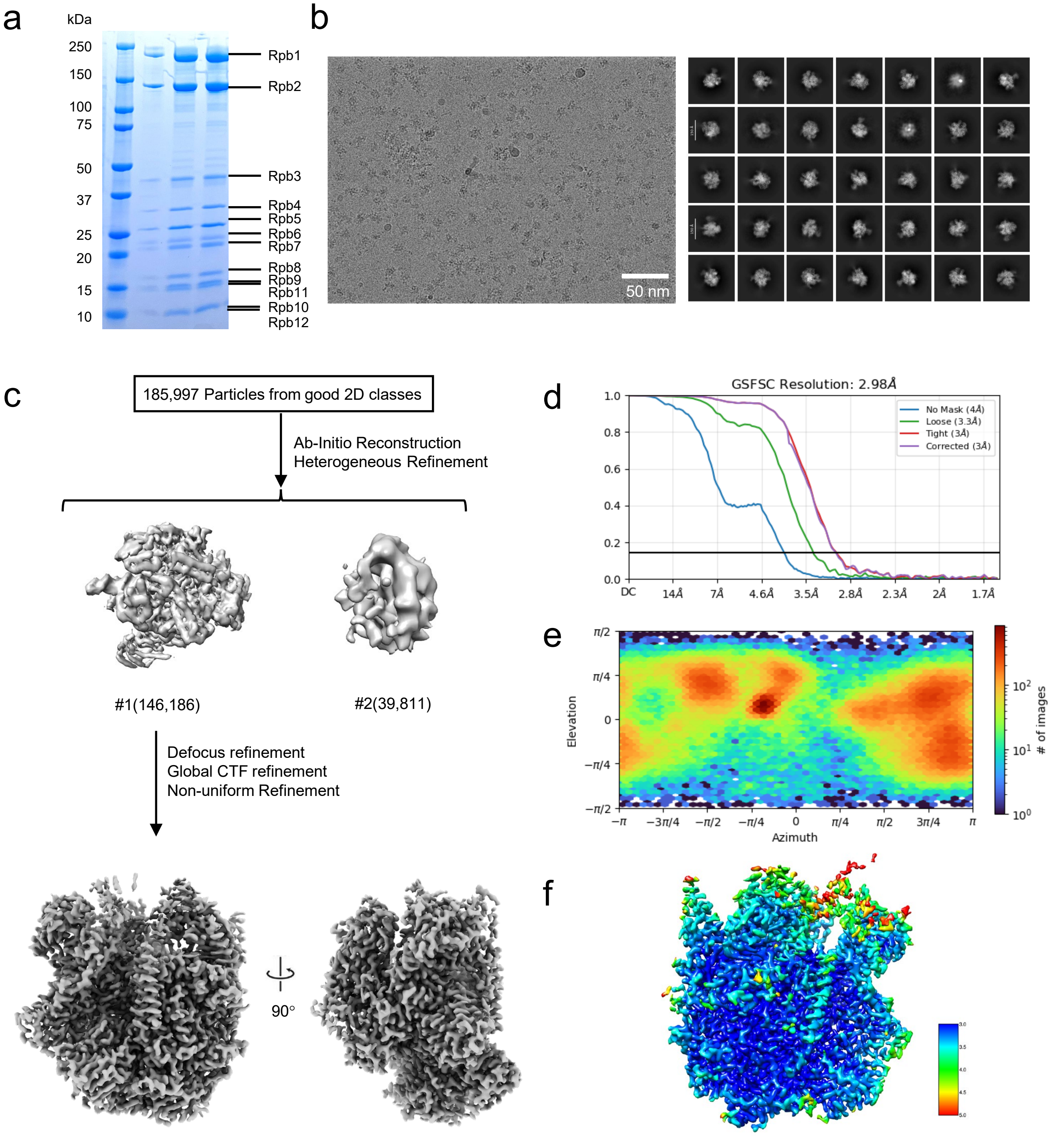

Supplementary Figure 12 | Data processing summary of scPolI EC complex captured by mspSA affinity grids. (a) SDS-PAGE analysis of scPolI EC complex after SEC. **(b)** Rrepresentative raw micrograph (top) and gallery of reference-free 2D class averages (bottom) of scPolI EC complex. **(c)** Workflow of scPolI EC complex structure determination. **(d-f)** Corrected FSC **(d)**, orientational distribution heat map **(e)** and local resolution **(f)** of the density map of scPolI EC complex.

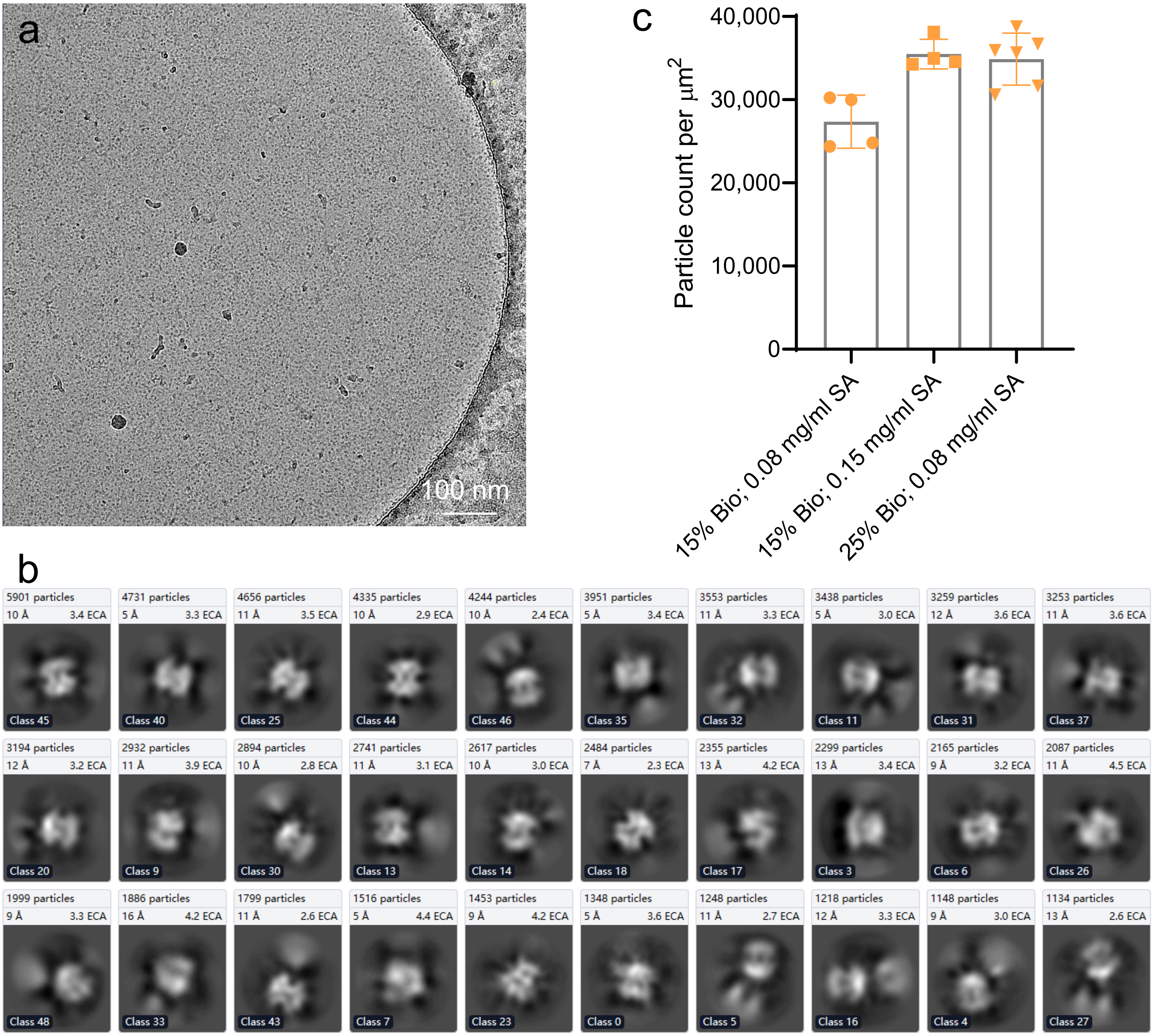

Supplementary Figure 13 | Identification and analysis of the SA particles. (a) A typical micrograph of the SA. The experiment was repeated at least three times independently with similar results. **(b)** Typical 2D classes of the SA particles. The SA particles were identified by 2D averageing function in CryoSPARC. **(c)** The number of SA particles per μm^2 at a series of conditions. The error bars are standard deviation.
