# Supplementary material for: Open architecture of archaea MCM and dsDNA complexes resolved using monodispersed streptavidin affinity CryoEM
Source: Nat Commun. 2024 Nov 27;15:10304. doi: 10.1038/s41467-024-53745-w (PMC11603195; doi:10.1038/s41467-024-53745-w)
Supplement: Supplementary file 2 — Description of Additional Supplementary Files [file 41467_2024_53745_MOESM2_ESM.pdf]

Description of Additional Supplementary Files

File name: Supplementary Movie 1
Description: A 360° perspective of the fitting of a dsDNA into the segmented density (orange).
