# Supplementary material for: Open architecture of archaea MCM and dsDNA complexes resolved using monodispersed streptavidin affinity CryoEM
Source: Nat Commun. 2024 Nov 27;15:10304. doi: 10.1038/s41467-024-53745-w (PMC11603195; doi:10.1038/s41467-024-53745-w)
Supplement: Supplementary file 4 — Reporting Summary [file 41467_2024_53745_MOESM4_ESM.pdf]

nature research

Corresponding author(s): Peijun Zhang

Last updated by author(s): Sep 30, 2024

Reporting Summary

Nature Research wishes to improve the reproducibility of the work that we publish. This form provides structure for consistency and transparency in reporting. For further information on Nature Research policies, see Authors & Referees and the Editorial Policy Checklist.

Statistics

For all statistical analyses, confirm that the following items are present in the figure legend, table legend, main text, or Methods section.

n/a	Confirmed	
☐	☒	The exact sample size (n) for each experimental group/condition, given as a discrete number and unit of measurement
☐	☒	A statement on whether measurements were taken from distinct samples or whether the same sample was measured repeatedly
☒	☐	The statistical test(s) used AND whether they are one- or two-sided<br>*Only common tests should be described solely by name; describe more complex techniques in the Methods section.*
☒	☐	A description of all covariates tested
☒	☐	A description of any assumptions or corrections, such as tests of normality and adjustment for multiple comparisons
☐	☒	A full description of the statistical parameters including central tendency (e.g. means) or other basic estimates (e.g. regression coefficient) AND variation (e.g. standard deviation) or associated estimates of uncertainty (e.g. confidence intervals)
☒	☐	For null hypothesis testing, the test statistic (e.g. F, t, r) with confidence intervals, effect sizes, degrees of freedom and P value noted<br>*Give P values as exact values whenever suitable.*
☒	☐	For Bayesian analysis, information on the choice of priors and Markov chain Monte Carlo settings
☒	☐	For hierarchical and complex designs, identification of the appropriate level for tests and full reporting of outcomes
☒	☐	Estimates of effect sizes (e.g. Cohen's d, Pearson's r), indicating how they were calculated

Our web collection on statistics for biologists contains articles on many of the points above.

Software and code

Policy information about availability of computer code

Data collection	EPU, FEI TIA
Data analysis	MotionCor2, CryoSPARC3.3.2, AlphaFold2, UCSF Chimera 1.14, UCSF ChimeraX 1.5, COOT 0.981, PHENIX 1.14, MolProbity.

For manuscripts utilizing custom algorithms or software that are central to the research but not yet described in published literature, software must be made available to editors/reviewers. We strongly encourage code deposition in a community repository (e.g. GitHub). See the Nature Research guidelines for submitting code & software for further information.

Data

Policy information about availability of data

All manuscripts must include a data availability statement. This statement should provide the following information, where applicable:

- Accession codes, unique identifiers, or web links for publicly available datasets
- A list of figures that have associated raw data
- A description of any restrictions on data availability

All data needed to evaluate the conclusions in the paper are present in the paper and/or the Supplementary information and source data are provided with this paper. The cryo-EM density maps and corresponding atomic models have been deposited in the EMDB and PDB, respectively. The accession codes are: for MCM-apo, EMD-38109 and PDB 8X7T [https://doi.org/10.2210/pdb8X7T/pdb] (the composite map), EMD-38110 (the map before local refinement), EMD-38112 to EMD-38118 (maps after local refinement); for MCM-ATP-dsDNA: EMD-38111 and PDB 8X7U [https://doi.org/10.2210/pdb8X7U/pdb] (the composite map), EMD-38119 (the map before local refinement), EMD-38120 to EMD-38127 (maps after local refinement); for CA Di-hexamer EMD-61286 and PDB 9JA0 [https://doi.org/10.2210/pdb9JA0/pdb]; for ScPol II EC: EMD-61287 and PDB 9JA1 [https://doi.org/10.2210/pdb9JA1/pdb].

Field-specific reporting

Please select the one below that is the best fit for your research. If you are not sure, read the appropriate sections before making your selection.

☒ Life sciences ☐ Behavioural & social sciences ☐ Ecological, evolutionary & environmental sciences

For a reference copy of the document with all sections, see nature.com/documents/nr-reporting-summary-flat.pdf

Life sciences study design

All studies must disclose on these points even when the disclosure is negative.

Sample size	For cryo-EM SPA, sample sizes were those required for the resolution. The details of datasets, including sample sizes, are listed in the Supplementary Table 3 and 4.
Data exclusions	During cryo-EM SPA study, the particles were autopicked by a density threshold, which is a standard approach in cryoSPARC. 2D and 3D classifications were preformed to remove the junk particles in order to generate higher-resolution density maps.
Replication	For cyro-EM SPA study, two randomly divided half datasets were processed independently and combined to give rise to the final structures. The resolution of the structure is assessed by comparing the two independent maps.
Randomization	For each dataset of Cryo-EM SPA study, the dataset were randomly divided into two half sets, as a standard approach implementd in cryoSPARC.
Blinding	The cryo-EM SPA study including data collection and processing is not blinded.

Reporting for specific materials, systems and methods

We require information from authors about some types of materials, experimental systems and methods used in many studies. Here, indicate whether each material, system or method listed is relevant to your study. If you are not sure if a list item applies to your research, read the appropriate section before selecting a response.

Materials & experimental systems

n/a	Involved in the study
☒	☐ Antibodies
☒	☐ Eukaryotic cell lines
☒	☐ Palaeontology
☒	☐ Animals and other organisms
☒	☐ Human research participants
☒	☐ Clinical data

Methods

n/a	Involved in the study
☒	☐ ChIP-seq
☒	☐ Flow cytometry
☒	☐ MRI-based neuroimaging
